# Effects of pH and Mineral Nutrition on Growth and Physiological Responses of Trembling Aspen (*Populus tremuloides*), Jack Pine (*Pinus banksiana*), and White Spruce (*Picea glauca*) Seedlings in Sand Culture

**DOI:** 10.3390/plants9060682

**Published:** 2020-05-27

**Authors:** Feng Xu, Maryamsadat Vaziriyeganeh, Janusz J. Zwiazek

**Affiliations:** Department of Renewable Resources, University of Alberta, 442 Earth Sciences Building, Edmonton, AB T6G 2E3, Canada; fx1@ualberta.ca (F.X.); vaziriye@ualberta.ca (M.V.)

**Keywords:** pH tolerance, mineral nutrition, sand culture, tree seedlings, gas exchange, growth, oil sands revegetation

## Abstract

Responses of trembling aspen (*Populus tremuloides*), jack pine (*Pinus banksiana*), and white spruce (*Picea glauca*) seedlings to root zone pH ranging from 5 to 9 were studied in sand culture in the presence of two mineral nutrition levels. After eight weeks of treatments, effects of pH on plant dry weights varied between the plant species and were relatively minor in white spruce. Higher nutrient supply significantly increased dry weights only in trembling aspen subjected to pH 5 treatment. There was little effect of pH and nutrition level on net photosynthesis and transpiration rates in white spruce and jack pine, but net photosynthesis markedly declined in aspen at high pH. Chlorophyll concentrations in young foliage decreased the most in trembling aspen and jack pine. The effects of high pH treatments on the concentrations of Mg, P, Ca, Mn, Zn, and Fe in young foliage varied between the plant species with no significant decreases of Fe and Zn recorded in trembling aspen and white spruce, respectively. This was in contrast to earlier reports from the studies carried out in hydroponic culture. The sand culture system that we developed could be a more suitable alternative to hydroponics to study plant responses to pH in the root zone. Plant responses to high pH appear to involve complex events with a likely contribution of nutritional effects and altered water transport processes.

## 1. Introduction

Soil pH affects plant growth through its complex interactions involving changes in soil chemistry and physical properties that impact physiological processes in plants [1]. More than 30% of the world’s soils have a high pH problem including vast areas of calcareous, saline, and sodic soils [2]. Plants vary in their soil pH optimum. Natural undisturbed ecosystems have evolved over time, and the plants in these ecosystems are adapted to specific soil pH conditions. However, in agricultural areas and in other places affected by human activities, plants may be exposed to soil pH that is outside of their tolerance range. Soil disturbance that results in rapid and dramatic changes of soil pH is especially challenging to plants. In northeastern Alberta, Canada, oil sands mining has disturbed large areas of the boreal ecosystems [3]. These areas must be restored with local vegetation to the pre-disturbance equivalent land capability [4]. However, in the oil sands reclamation areas, soil pH frequently exceeds 8.0, while the pH of undisturbed soils in the surrounding boreal forests is typically below 6.0 [5].

Since high pH decreases the availability of Mg, Ca, Fe, Mn, P, Zn, and other mineral nutrients in soil solution [6,7], the main effects of high soil pH on plants are frequently attributed to nutrient deficiencies [8]. Under nutrient deficiency conditions, plant response strategies include increased nutrient use and acquisition efficiencies [8]. Plant apoplastic pH is approximately 5.5, while the cytoplasm pH is about 7.2–7.4 [9]. The high soil pH increases the root apoplastic pH, thereby impairing the pH gradient across the plasma membrane, which is essential for nutrient uptake [10].

Plant roots can lower the rhizosphere pH by pumping protons as one of the main mechanisms of high soil pH tolerance [1,11,12]. However, due to highly variable properties and commonly high buffering capacity of soil, controlled-environment studies aimed at understanding the effects and mechanisms of action of root zone pH on plants are limited and have been mostly carried out in hydroponic culture [13,14,15,16]. Hydroponic systems with nutrient solution circulating around plant roots poorly reflect the dynamics that are present in the solid medium [17] and practically eliminate the effectiveness of proton extrusion. Hydroponic culture also alters root structure in many plants [18] and affects other morphological and physiological properties [19]. Therefore, the uptake and transport of nutrients and water may vary in hydroponic and soil-grown plants.

In addition to nutrient uptake, numerous studies have pointed to the inhibition of root cell elongation and root water transport among the principal mechanisms contributing to plant growth reductions in plants exposed to high root zone pH [13,14,20,21,22]. An inability of plants to maintain root apoplast acidification can affect cell elongation and impair root growth [13,22,23,24]. High pH also reduces root water flux [14,25] and results in reductions in shoot water potential [13,21] and transpiration rates [7,14,20]. However, relative contributions of mineral nutrition and water relations to growth responses in plants affected by high pH are difficult to separate.

Since plant responses to root zone pH may be affected by the root growth medium, we developed for the present study a semi-automated sand culture system to examine the effects of pH and mineral nutrition levels on growth and physiological responses in trembling aspen (*Populus tremuloides*), jack pine (*Pinus banksiana*), and white spruce (*Picea glauca*) seedlings, which are commonly used for oil sands reclamation with elevated soil pH. The main objective of this study was to examine the effects of different root zone pH levels and nutrient supply in sand culture to develop a better understanding of the mechanisms of high pH tolerance and assess the suitability of these plant species for the reclamation of oil sands mining areas. Despite their commercial and ecological importance, soil pH tolerance of these tree species has been rarely studied. Additionally, since most of the previous studies examined the effects of high pH on plants in the presence of other confounding factors such as salinity [15,26], high pH tolerance mechanisms in the absence of these confounding factors are little understood.

The main objective of the study was to compare the responses of the three boreal plant species to root zone pH in sand culture. In controlled-environment hydroponic studies, white spruce exhibited relatively high tolerance, while trembling aspen and jack pine were moderately tolerant to high root zone pH [7,16,21,22]. Due to higher growth rates and greater nutrient demand of trembling aspen compared with the two studied conifer species, we hypothesized that increased nutrient supply would be more effective in ameliorating the high pH effects on trembling aspen compared with white spruce and jack pine.

## 2. Results

### 2.1. Total Dry Weights and Shoot to Root (s/r) Dry Weight Ratios

**Figure 1:**
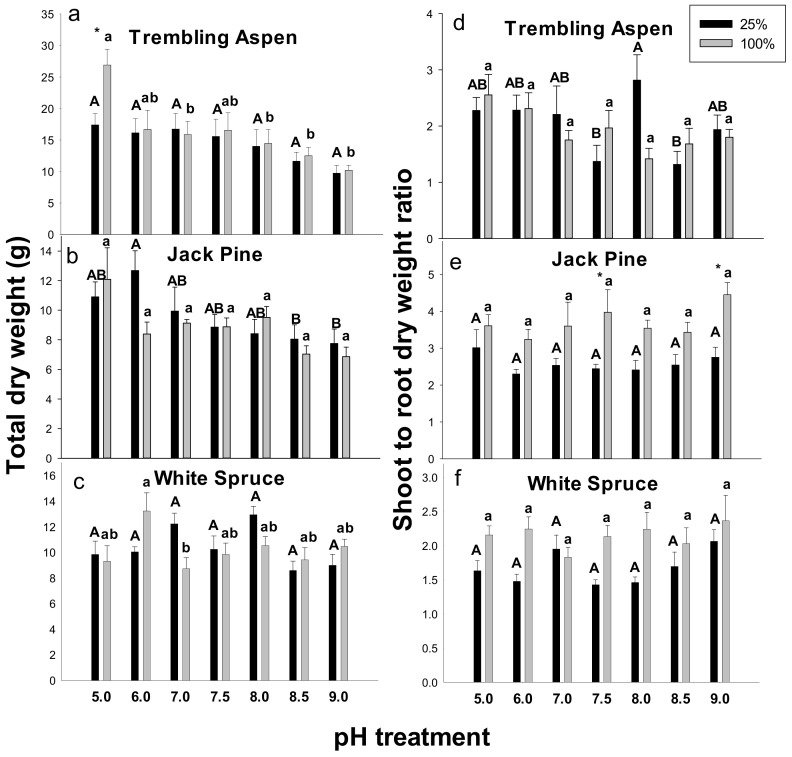
Effects of pH treatments and nutrition level (supplied with 25% and 100% Hoagland’s solution) on the total dry weights (**a**–**c**) and shoot to root dry weight ratios (**d**–**f**) in trembling aspen (**a**,**d**), jack pine (**b**,**e**), and white spruce (**c**,**f**). Different letters above the bars (uppercase letters for 25% Hoagland’s solution and lowercase letters for 100% Hoagland’s solution) indicate significant differences (α = 0.05) between pH treatments within each plant species. The asterisk above the bars indicates significant differences (α = 0.05) between 25% and 100% Hoagland’s solution. Means (*n* = 8) ± SE are shown.

Total dry weights of trembling aspen supplied with 100% Hoagland’s solution were higher by over 50% compared with the plants supplied with 25% Hoagland’s solution at pH 5.0 (Figure 1a). At the 25% Hoagland’s solution level, there was no significant effect of pH on the total dry weights in trembling aspen (Figure 1a).

There was no significant effect of the Hoagland’s solution concentration on the total dry weights at any of the examined pH levels in jack pine (Figure 1b) and white spruce (Figure 1c). In jack pine, lower total dry weights were observed at pH 8.5 and 9.0 compared with pH 5.0 in 25% Hoagland’s solution (Figure 1b). In white spruce, the total dry weights were lower at pH 7.0 compared with pH 5.0 in 100% Hoagland’s solution (Figure 1c). The interaction effects (nutrition x pH) on the total dry weight were significant in white spruce (Table S1).

In trembling aspen, the interaction of pH and Hoagland’s solution concentration on s/r dry weight ratios was significant (Table S1), and the highest s/r ratios in trembling aspen were measured at pH 8.0 in 25% Hoagland’s solution (Figure 1d). In jack pine, the s/r ratios at pH 7.5 and 9.0 in 100% Hoagland’s solution were higher by about 80% compared with 25% Hoagland’s solution at the same pH (Figure 1e). The pH level and Hoagland’s solution concentration had little effect on s/r ratio in white spruce (Figure 1f).

### 2.2. Gas Exchange

**Figure 2:**
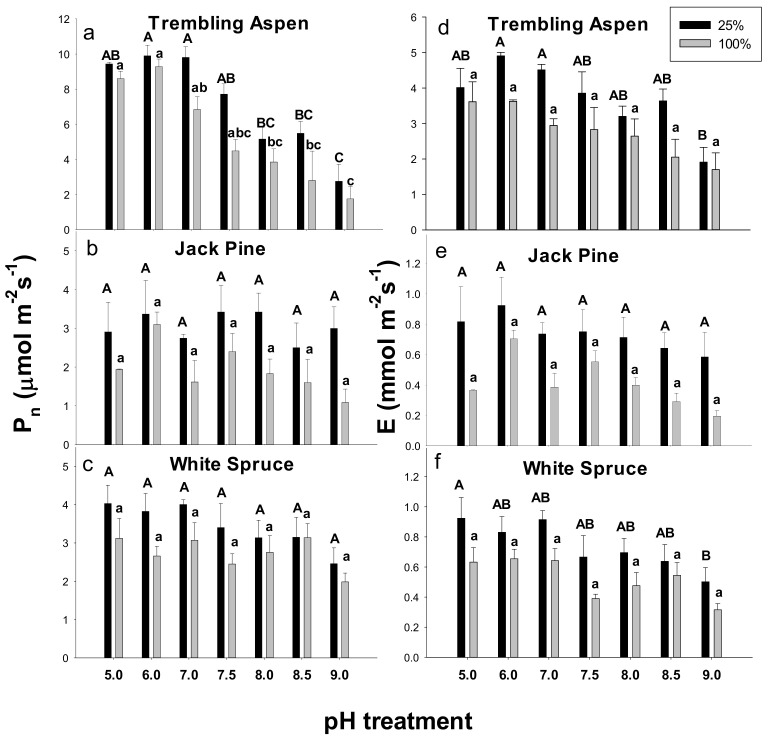
Effects of pH treatments and nutrition level (supplied with 25% and 100% Hoagland’s solution) on net photosynthesis (Pn) (**a**–**c**) and transpiration rates (E) (**d**–**f**) in trembling aspen (**a**,**b**), jack pine (**c**,**d**), and white spruce (**e**,**f**). Different letters above the bars (uppercase letters for 25% Hoagland’s solution and lowercase letters for 100% Hoagland’s solution) indicate significant differences (α = 0.05) between pH treatments within each plant species. Means (*n* = 8) ± SE are shown.

There were no significant interaction effects (nutrition × pH) for both photosynthesis (Pn) and transpiration rate (E) in the three examined species (Table S1). In trembling aspen, significant reductions of Pn occurred at pH 8.0–9.0 in both concentrations of Hoagland’s solution (Figure 2a). There were no significant differences in Pn across the pH and Hoagland’s solution levels (Figure 2b,c). In trembling aspen, E decreased at pH 9.0 in 25% Hoagland’s solution compared with pH 5–7 (Figure 2d). In jack pine, E showed a consistently lower trend in 100% compared with 25% Hoagland’s solution at all pH levels. However, the differences were not statistically significant when compared at each pH (Figure 2e,f). In white spruce, E was significantly lower in the 25% Hoagland’s solution at pH 9.0 compared with pH 5.0 (Figure 2f).

### 2.3. Chlorophyll Concentrations

**Figure 3:**
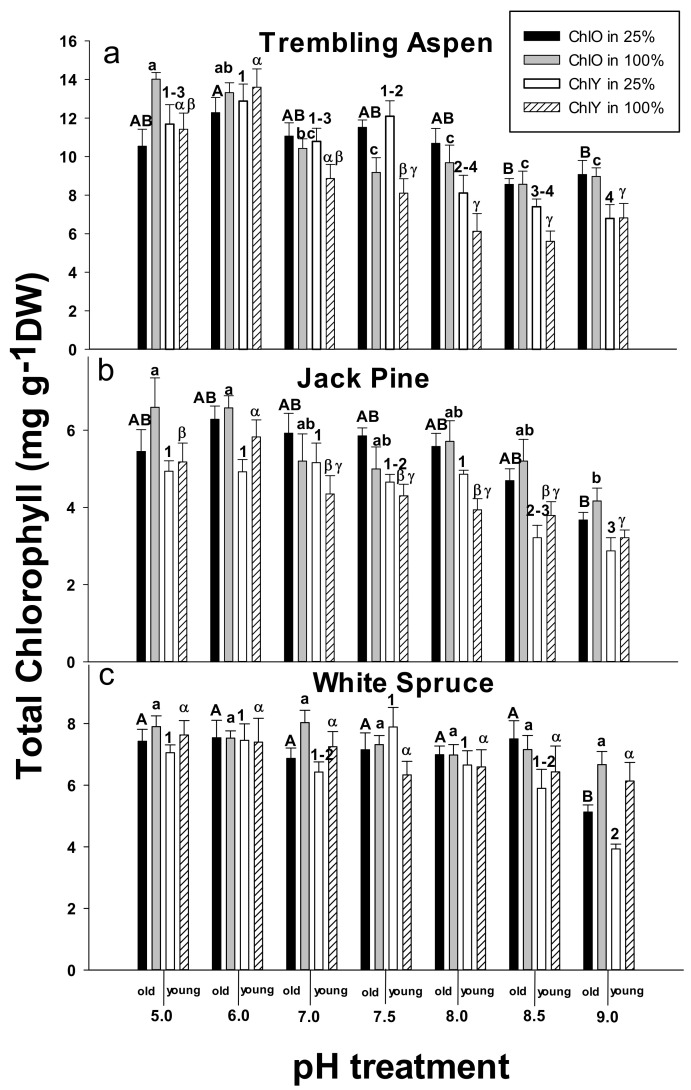
Effects of pH and nutrition level on chlorophyll concentrations in old (ChlO) and young (ChlY) foliage of trembling aspen (**a**), jack pine (**b**), and white spruce (**c**). Different letters and numbers above the bars (uppercase letters for ChlO in 25% Hoagland’s solution, lowercase letters for ChlO in 100% Hoagland’s solution, numbers for ChlY in 25% Hoagland’s solution, and Greek letters for ChlY in 100% Hoagland’s solution) indicate significant differences (α = 0.05) between treatments within each plant species. Means (*n* = 6) ± SE are shown.

Decreased foliar chlorophyll concentrations at high pH were observed in the three plant species at both Hoagland’s solution levels (Figure 3a–c). There were significant interactions between pH and Hoagland’s solution levels for the chlorophyll concentrations in old leaves (ChlO) of trembling aspen, and for the chlorophyll concentrations in young needles (ChlY) of jack pine and white spruce (Table S1). In white spruce, both ChlO and ChlY were drastically reduced at pH 9.0 in the 25% Hoagland’s solution, while there was no significant pH effect in the 100% Hoagland’s solution (Figure 3c).

### 2.4. Elemental Concentrations of Young Leaves in 25% Hoagland’s Solution

**Figure 4:**
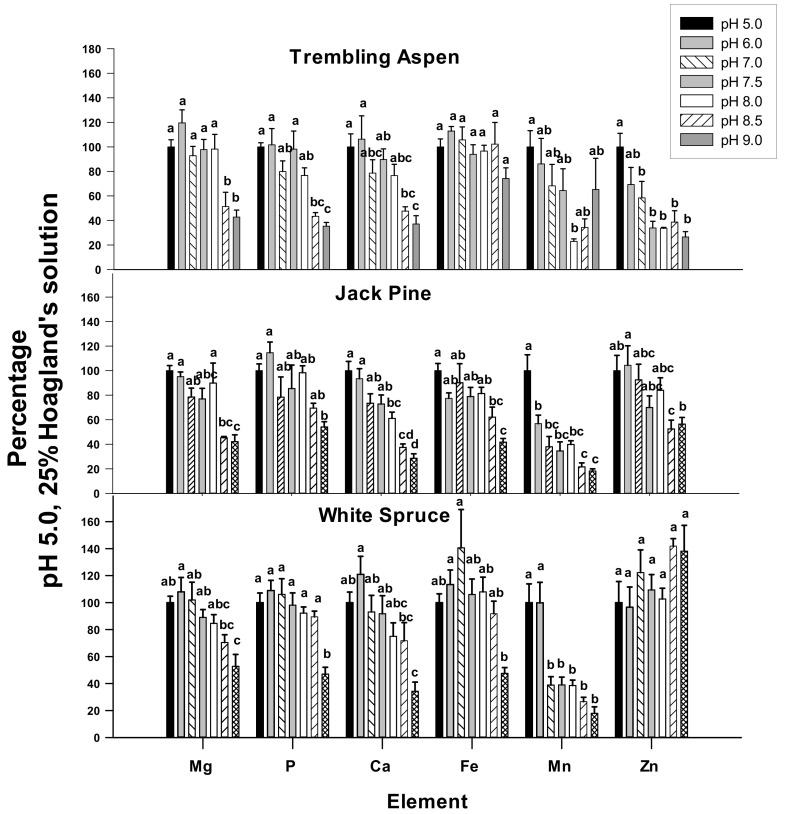
Effects of pH on Mg, P, Ca, Fe, Mn, and Zn concentrations in young foliage of plants supplied with 25% Hoagland’s solution, presented as the percentages of values measured at pH 5.0. Different letters above the bars indicate significant differences (α = 0.05) between treatments within each plant species. Means (*n* = 6) ± SE are shown.

In trembling aspen, the concentrations of Mg, P, Ca, Mn, and Zn decreased in young leaves when the roots were exposed to high pH (Figure 4). The concentrations of Mg, P, and Ca decreased at pH 8.5 and 9.0 while Zn decreased with pH of 7.0 and higher (Figure 4). The pH treatments had no significant effects on Fe concentrations in trembling aspen (Figure 4).

There were significant reductions in Mg, P, Ca, Mn, Zn, and Fe in young needles at high pH in jack pine. The pH threshold required to trigger these decreases varied depending on the element. For Mg, P, Ca, Fe, and Zn, the decreases occurred at pH 8.0 and higher. However, the concentration of Mn decreased at and above pH 6.0 (Figure 4).

In white spruce, the concentrations of Mg, P, Ca, and Fe decreased at pH 9.0, but the concentrations of Mn decreased at and above pH 7.0 (Figure 4). There was no significant effect of pH on Zn concentrations in young white spruce needles (Figure 4).

## 3. Discussion

Studies on the effects of root zone pH on plants have been commonly carried out in hydroponic culture due to the difficulties with effective pH control [13,16,21,22]. For the present investigation, we developed a semi-automated sand culture system to examine physiological responses in seedlings of three boreal tree species to root zone pH as affected by the supply of mineral nutrients in the solid medium. Using this system, we were able maintain the sand pH within ± 0.5 of the pre-set levels over the two-month period of the study.

We selected three species of boreal trees, including jack pine (*Pinus banksiana*), trembling aspen (*Populus tremuloides*), and white spruce (*Picea glauca*), since they are used for the revegetation of oil sands mining areas that are commonly affected by high soil pH [27]. Compared with white spruce and jack pine, trembling aspen was earlier found to respond more strongly with nutrient deficiencies at high pH in hydroponic culture [16]. However, in the present study, despite higher growth rates and greater nutrient demand of trembling aspen, compared with relatively slow-growing white spruce and jack pine seedlings, with the exception of Zn and P, high pH did not appear to have a clearly greater effect on the concentrations of the examined elements compared with the two examined conifer species. On the contrary, the concentrations of Mn in young needles of white spruce and jack pine decreased more at high pH compared with young leaves of aspen. Interestingly, contrary to the results reported for hydroponic culture [21,28], Fe foliar concentrations were not significantly affected by pH in trembling aspen, but decreased at pH 9.0 in white spruce and at pH 8.5 and 9.0 in jack pine. The results demonstrate species differences in Fe uptake or translocation to new foliage at high pH as previously demonstrated for different species of boreal trees [16,21,28].

Except for pH 5.0 in trembling aspen, 25% and 100% concentrations of Hoagland’s mineral solution had relatively little effect on the total dry weights in the three examined species. It is interesting that the higher total dry weights in trembling aspen that were provided with 100% Hoagland’s solution at pH 5.0 were not accompanied by higher Pn. Also, it was surprising to find a sharp increase in s/r ratios in trembling aspen at pH 8.0 and 35% Hoagland’s solution, which may reflect the pH-dependent nutrient availability dynamics and their consequences on C allocation. Increased nutrient supply may enhance leaf production with no increases of Pn, which can result in an overall higher plant photosynthesis due to greater photosynthetic area [29,30]. In the present study, significant pH reductions in Pn of aspen were measured at pH 8.0–9.0, and the only significant effect on E was in the 25% Hoagland’s solution treatment at pH 9.0. However, there was an overall decreasing trend in E at high pH levels that varied between the plant species. Although the applied pH treatments resulted in slightly different pH levels in the hydroponic and sand media, trembling aspen responses in sand culture sharply contrasted with those reported for hydroponic culture, in which total dry weights and gas exchange were drastically reduced at and above pH 7.5 [16,21]. The water uptake dynamics are likely to be different in plants growing in hydroponics and solid medium, and the effects of pH on E are likely a reflection of the pH effect on root water transport properties [14,22].

Total dry weights and gas exchange parameters in jack pine were little affected by the pH treatments in sand culture. There was also little effect of pH on the total dry weights, s/r ratios, and gas exchange parameters in white spruce, except for the significant E decrease in 25% Hoagland’s solution treatment at pH 9.0 compared with pH 5.0. Jack pine was demonstrated to be moderately sensitive [16,22], while white spruce was relatively tolerant of high pH in hydroponic culture [16,21]. Therefore, growth and gas exchange responses to pH may vary between the plant species in hydroponic and sand cultures.

Plant roots can mediate rhizosphere pH in response to environmental constraints [11]. H^+^-ATPases play an important role as they modify the rhizospheric pH while playing a fundamental role in nutrient uptake [1]. Interacting with a rhizosphere, the apoplastic pH of roots could be lower than that of the growth medium. In lupin (*Lupinus angustifolius* L.), the root apoplastic pH increased by 0.3 units when the external root zone pH increased from 5.2 to 7.5 [31]. However, in hydroponic culture, the constant circulation of nutrient solution makes it more difficult for plants to maintain a proton gradient compared with the solid growth medium.

The effects of pH and mineral nutrition levels on foliar chlorophyll concentrations varied between the three studied plant species. While there were significant interactions between pH and Hoagland’s solution levels for the chlorophyll concentrations in older leaves of trembling aspen, the interactions were significant for younger needles in jack pine and white spruce, suggesting that different factors could be responsible for the decreases in chlorophyll concentrations. In jack pine, needle chlorophyll concentrations were found to decrease at root zone pH ≥ 7.0, and with the exception of pH 9.0, these decreases occurred in the absence of clear deficiencies of the analyzed essential elements in the tissues [22].

Similarly to growth and gas exchange, high pH can trigger changes in foliar chlorophyll concentrations through both chlorophyll synthesis and degradation processes. In high pH environments, nutrient deficiencies including Mg [32], Fe [33], and Mn [34] affect chlorophyll synthesis. Therefore, we expected that the higher nutrition level would have a positive effect on foliar chlorophyll concentrations. However, with the exception of white spruce at pH 9.0, we did not see a clear effect of the 100% compared with 25% Hoagland’s solution treatment. Leaf chlorosis is a complex response that can be affected by various stress factors. In hydroponic culture, reduced chlorophyll concentrations, root growth, and gas exchange were correlated with an inhibition of water uptake at high pH [22].

Although under nutrient deficiency stress, photosynthetic depression was found to be caused by the biochemical, rather than stomatal, limitation [35], we observed a decreasing trend in E with increasing treatment pH. The reductions of E by the high pH treatments likely reflect altered water balance due to reduced water delivery to the leaves. Root hydraulic conductance is generally reduced at high pH leading to stomatal closure [14,36]. The decrease in root water flux may result from the reduction of root aquaporin activity [14] and (or) the reduced root system size [22,37]. However, in our study, we did not see a clear effect of sand culture pH on the shoot-to-root ratios in the examined plants. Our results suggest that the reductions in Pn in trembling aspen could be due to a combination of high pH nutritional effects, foliar chlorophyll concentrations, as well as altered water balance, possibly, due to the effects on water transport.

## 4. Materials and Methods

### 4.1. Plants and Experimental Setup

One-year-old dormant seedlings of trembling aspen (*Populus tremuloides* Michx.), jack pine (*Pinus banksiana* Lamb.), and white spruce (*Picea glauca* (Moench) Voss) were obtained from the Boreal Horticultural Services Ltd. (Bonnyville, Alberta, Canada). The seedlings had been grown in the tree nursery from seed in containers (415D Styroblocks™, Beaver Plastics, Acheson, AB, Canada) for one year. After the roots of seedlings had been washed free of soil, the seedlings were transplanted into 4.5 L pots filled with washed sand (10/20 filter sand and 20/40 abrasive sand 3:1 (v/v), Target Products Ltd., Burnaby, BC, Canada). Garden fabric (Spectrum brands Inc., Madison, WI, USA) was placed at the bottom of the pots to prevent leaking of the sand. The plants were grown in a controlled environment growth room at 22/18 °C (day/night) temperature, 65% ± 10% relative humidity, and 16 h photoperiod with 300 μmol m^−2^ s^−1^ photosynthetic photon flux density (PPFD). The plants were supplied with 25% modified Hoagland’s mineral solution [9] twice a week for 3 weeks before the commencement of treatments. The composition of modified 100% strength Hoagland’s solution is shown in Table S2.

An automated irrigation system was set up for this study to maintain uniform water supply and stable pH. The system consisted of two separate components, the first one controlling the delivery rate of nutrient solution and the second one maintaining the desirable pH in the sand. For the watering and nutrient delivery system, Hoagland’s solutions were in seven 120 L polyvinyl chloride (PVC) drums (seven drums with 25% and seven drums with 100% Hoagland’s solution) and delivered to each pot by a water pump (Model 9.5 950GPH, Danner MFG Inc., New York, NY, USA) through a tubing setup. The main part of the tubing setup was the 19 mm PVC tubing to which 6 mm PVC tubing was connected and attached to the top of each pot by the 4 x 6 mm support stakes. There were four emitters connected to the 6 mm tubing to ensure that the same volume of solution was delivered to each pot. A timer was connected to the water pumps to control the watering schedule. In the pH control system, a gel-filled combination pH electrode (Orion 9106 BNWP, Thermo Scientific, Rochester, NY) was placed in each drum with Hoagland’s solution and connected to a pH controller (PHCN-70, Omega Engineering Inc., Laval, QC, Canada), which controlled an electronic valve (Model 8260G071 120/60 ASCO Valve, Inc., Florham Park, NJ, USA). The valve opened and closed to adjust the solution pH to the pre-set level by adding 5% (w/w) KOH or 1% (v/v) H_2_SO_4_.

### 4.2. Experimental Treatments

**Figure 5:**
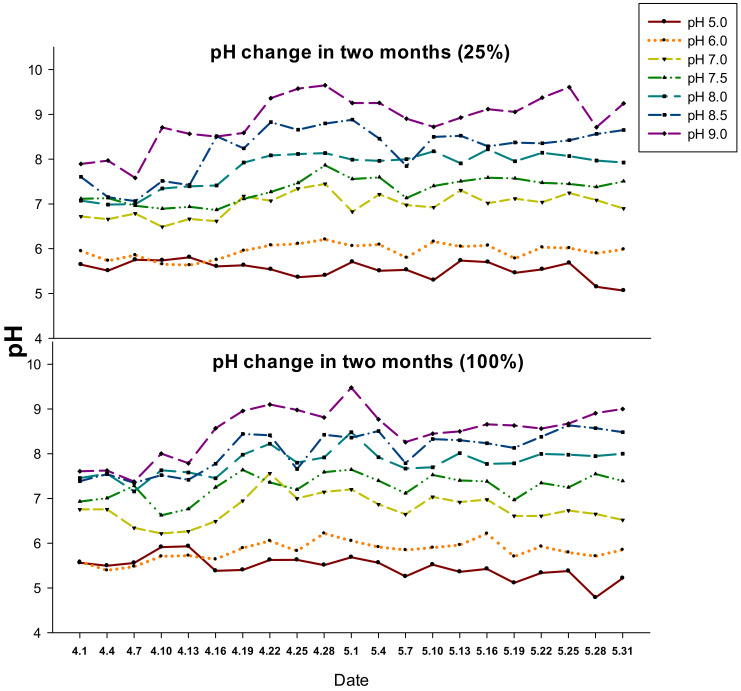
Changes in sand pH during the two months of treatments with 25% and 100% Hoagland’s solution with the pH levels preset at day 0 to 5.0–9.0.

The pots with seedlings were randomly placed on a bench in a growth room, and the seedlings were subjected to different nutrition and pH treatments for eight weeks. There were eight seedlings per treatment for a total of 112 plants for each plant species. The treatments consisted of two nutrition levels (25% and 100% Hoagland’s solution) and seven pH levels (5.0, 6.0, 7.0, 7.5, 8.0, 8.5, and 9.0). The solution pH that was required to achieve a desired pH in the sand culture was experimentally determined in preliminary experiments (Table S3). The seedlings were provided with Hoagland’s solution at different pH levels three times per day. The sand pH was measured in four pots per treatment with a pH meter (Model IQ170, Hach Company Loveland, CO, USA.) equipped with a stainless steel probe (Model PH77-SS, Hach Company, London, ON, Canada) over the course of the experiment. The pH measurements were carried out twice a week, and the sand was flushed with deionized water every two weeks. The sand pH fluctuated over the course of treatments within ± 0.5 of the set levels (Figure 5).

### 4.3. Dry Weights and Foliar Chlorophyll Concentrations

After eight weeks of treatments, shoot and root dry weights were measured in eight seedlings (*n* = 8) per treatment for each tree species. Roots and shoots were separated and dried in an oven at 70 °C for 72 h. The leaves for chlorophyll measurements were detached from the stems and immediately placed in a freezer at −80 °C for 72 h. The leaves were separated into young leaves (those that emerged after the start of treatments) and old leaves (those that fully expanded before the treatments). The sum of the dry weights of stems, old leaves, and young leaves from each plant was referred to as the shoot dry weight.

Chlorophyll a and b concentrations were determined in fully expanded mature leaves (needles) and young expanding leaves (needles) in six randomly selected seedlings per treatment (*n* = 6) for each species. After freeze-drying, the leaves (needles) were ground with a Thomas Wiley Mini-Mill (Thomas Scientific, NJ, USA). Pulverized leaf samples (10 mg) were extracted with 8 mL dimethylsulfoxide (DMSO) at 65 °C for 22 h. Chlorophyll concentrations were measured in DMSO extracts at 648 and 665 nm for chlorophyll a and b with a spectrophotometer (Ultrospec, Pharmacia LKB, Uppsala, Sweden). Total chlorophyll concentrations were calculated using the Arnon’s equation for DMSO [38].

### 4.4. Net Photosynthesis (Pn) and Transpiration (E) Rates

After eight weeks of treatments, Pn and E were measured in eight seedlings (*n* = 8) per treatment for each plant species. Fully expanded leaves with minimal or no necrosis were selected in the uppermost branches, and Pn and E were measured using the infrared gas analyzer (LI-6400, LI-COR, Lincoln, NE, USA). The reference CO_2_ concentration was 400 μmol mol^−1^, and the flow rate was 200 μmol s^−1^ in the leaf chamber. The leaf chamber temperature was kept at 20 °C, and the PPFD was set to 400 μmol m^−2^ s^−1^. The measurements were taken from 4 to 10 h after the onset of photoperiod. For conifers, about 3 cm distal parts of the uppermost branch in white spruce and about 3 cm distal parts of the needles in jack pine were placed in the leaf chamber for the measurements. The needles in the leaf chamber were then severed and scanned to determine needle areas with the Sigma-scan Pro 5.0 (Systat Software, San Jose, CA, USA).

### 4.5. Elemental Analysis of Young Foliage

Six seedlings (*n* = 6) were randomly selected per treatment from each plant species. Elemental concentrations were analyzed in young leaves (needles) of plants subjected to the different pH treatments in 25% Hoagland’s solution since visible symptoms, including leaf chlorosis, occurred mainly in these areas. Concentrations of Mg, P, Ca, Fe, Mn, and Zn were determined due to concerns of their possible reduced uptake at high pH [8,21,39]. Ground foliage samples of 0.3–0.4 g dry weight were digested with 10 mL 70% HNO_3_ and heated in a digestion block for 1 h. After complete digestion and cooling, the solution was diluted with Milli-Q water to 40 mL. The extracts were then filtered and analyzed by inductively coupled plasma mass spectrometry (ICP-MS) in the Radiogenic Isotope Facility at the University of Alberta, Edmonton, AB, Canada [40].

### 4.6. Experimental Design and Statistical Analysis

All data were analyzed with SAS (Version 9.3, SAS Institute Inc., Cary, NC) to determine statistically significant differences (*p* ≤ 0.05). The model was a complete randomized design with seven pH and two Hoagland’s solution levels. Two-way ANOVA was used to compare differences between the means. Residuals were checked for normality and homogeneity of variance. The log_10_ function was used to transform the data if they did not meet the ANOVA assumptions. Comparisons between different treatment means were carried out by Tukey’s test.

## 5. Conclusions

In conclusion, the responses of the studied plants to pH and nutrition levels in sand culture varied between the species, and this variation was likely related to the combination of factors. The sand culture system that we developed for the study provided roots with a more natural growth medium compared with the hydroponic system and, as expected, affected plant responses to the applied pH compared to the results reported earlier for the hydroponic studies. Contrary to our hypothesis, increased nutrient supply was not effective in alleviating the effects of high pH on the measured growth parameters and physiological characteristics in any of the three studied plant species. Also contrary to the results reported earlier for hydroponic culture, Fe foliar concentrations were not significantly affected by pH in trembling aspen, but decreased at pH 9.0 in white spruce and at pH 8.5 and 9.0 in jack pine. Plant responses to the root zone pH appear to involve nutritional factors combined with complex events that likely involve water transport processes. Since the sand culture system that we presented in this study offers a more natural root environment compared with hydroponic culture, it should be considered for futures studies of plant responses to pH in the root zone.

## Supplementary Materials

The following are available online at https://www.mdpi.com/2223-7747/9/6/682/s1, Table S1: ANOVA table showing the effects of pH and mineral nutrition treatments on the measured parameters in trembling aspen, jack pine and white spruce seedlings, Table S2: Composition of 100% modified Hoagland’s solution used in the study, Table S3: The pH levels of 25% and 100% Hoagland’s solutions that were required to achieve the aimed initial pH in sand culture.
