# Supplementary material for: Effects of pH and Mineral Nutrition on Growth and Physiological Responses of Trembling Aspen (Populus tremuloides), Jack Pine (Pinus banksiana), and White Spruce (Picea glauca) Seedlings in Sand Culture
Source: Plants (Basel). 2020 May 27;9(6):682. doi: 10.3390/plants9060682 (PMC7356384; doi:10.3390/plants9060682)
Supplement: Supplementary file 1 [file plants-09-00682-s001.pdf]

Table S1. ANOVA table showing the effects of pH and mineral nutrition treatments on the measured parameters in trembling aspen, jack pine, and white spruce seedlings.

**Trembling aspen**						
**p-value**	**tdw**	**s/r ratio**	**Pn**	**E**	**ChlO**	**ChlY**
**nutri**	0.1435	0.5272	0.0002	<0.0001	0.8623	0.0015
**pH**	<0.0001	0.0478	<0.0001	0.0023	<0.0001	<0.0001
**Nutri x pH**	0.2456	0.031	0.7181	0.4015	0.0033	0.0993
**p-value**	**Mg**	**P**	**Ca**	**Fe**	**Mn**	**Zn**
**pH**	<0.0001	<0.0001	0.0011	0.1262	0.0433	<0.0001
**Jack pine**						
**p-value**	**tdw**	**s/r ratio**	**Pn**	**E**	**ChlO**	**ChlY**
**nutri**	0.1765	<0.0001	0.0033	0.0009	0.5763	0.8062
**pH**	0.0013	0.2198	0.2656	0.0712	<0.0001	<0.0001
**Nutri x pH**	0.1107	0.8615	0.8682	0.2563	0.3745	0.0215
**p-value**	**Mg**	**P**	**Ca**	**Fe**	**Mn**	**Zn**
**pH**	<0.0001	0.0039	<0.0001	0.0002	<0.0001	0.0061
**White spruce**						
**p-value**	**tdw**	**s/r ratio**	**Pn**	**E**	**ChlO**	**ChlY**
**nutri**	0.6937	<0.0001	<0.0001	<0.0001	0.0539	0.2199
**pH**	0.024	0.3273	0.0002	<0.0001	0.0005	0.0001
**Nutri x pH**	0.0089	0.0763	0.6236	0.8809	0.187	0.0355
**p-value**	**Mg**	**P**	**Ca**	**Fe**	**Mn**	**Zn**
**pH**	0.0002	<0.0001	0.0003	0.0066	<0.0001	0.1072

Abbreviations: tdw, total dry weight (n = 8); s/r ratio, shoot to root dry weight ratio (n = 8); Pn, net photosynthetic rate (n = 8); E, transpiration rate (n = 8); ChlO, chlorophyll concentrations in old leaves (n = 6); ChlY, chlorophyll concentrations in young leaves (n = 6); foliar elemental concentrations (n = 6).

Table S2. Composition of 100% modified Hoagland's solution used in the study.

Compound	Concentration
KNO_3	6.0 mM
$Ca(NO_3)_2 \cdot 4H_2O$	4.0 mM
$NH_4H_3PO_4$	2.0 mM
$MgSO_4 \cdot 7\ H_2O$	1.0 mM
KCl	8.46 μM
H_3BO_3	4.17 μM
$MnSO_4 \cdot H_2O$	0.29 μM
$ZnSO_4 \cdot 7H_2O$	0.33 μM
$CuSO_4 \cdot 5H_2O$	0.08 μM
H_3MoO_4	0.08 μM
Fe-EDTA	20 μM

Table S3. The pH levels of 25% and 100% Hoagland's solutions that were required to achieve the aimed initial pH in sand culture.

Aimed Sand pH	Solution pH (25%)	Solution pH (100%)
5.0	3.5	3.8
6.0	5.0	5.0
7.0	9.5	9.0
7.5	10.0	9.5
8.0	10.5	10.0
8.5	11.0	10.5
9.0	11.5	11.0
